# Pathological effect of infectious bronchitis disease virus on broiler chicken trachea and kidney tissues

**DOI:** 10.14202/vetworld.2020.2203-2208

**Published:** 2020-10-21

**Authors:** Ismael I. Hasan, Saad T. Rasheed, Nawar A. Jasim, Mohammed K. Shakor

**Affiliations:** 1 Department of Pathology and Poultry Diseases, College of Veterinary Medicine, University of Tikrit, Tikrit, Iraq; 2 Department of Public Health, College of Veterinary Medicine, University of Tikrit, Tikrit, Iraq; 3 Salah-Adin Educational Veterinary Hospital, Tikrit, Iraq

**Keywords:** infections bronchitis virus, kidney, serotypes, trachea

## Abstract

**Aim:**

This study aimed to investigate the pathological effects of the infectious bronchitis virus (IBV) on chicken trachea and kidney tissues and also desired to diagnose the virus genome using a molecular tool.

**Materials and Methods:**

Twenty trachea and kidney samples collected from one broiler farm contain 10,000 chickens at Tikrit city. The chickens showed signs of gasping and mortality (20%) at early ages (20 days old), the presence of IBV investigated using conventional reverse transcriptase-polymerase chain reaction technique with routine histopathological study to tracheal and renal tissue.

**Results:**

Postmortem lesion showed severe respiratory inflammation with abscesses at tracheal bifurcation lead to airway blog. Molecular results showed two genotypes of IBV, one of them not included in primer designer research. The histological study showed different stages of inflammation, degeneration, and necrosis to the renal and tracheal tissues.

**Conclusion:**

The respiratory and renal pathological effect of the virus responsible for the symptoms appeared on the affected chicks that caused mortality, with a high probability of presence of a new viral genotype added to the untranslated region.

## Introduction

Infectious bronchitis disease one of the viral diseases that affect poultry worldwide, causing economic losses, especially in small ages leading to growth retardation, loss of appetite, and mortality [1]. Chicken and other avian species are considered the natural host of the virus [2]. Infectious bronchitis is an air-born disease transmitted through direct and indirect routes [3]. Infections bronchitis virus (IBV) belongs to the Coronaviridae family is a single positive-sense RNA consist of about 27 Kb encoding to four structural proteins named nucleocapsid (N), membrane (M), envelope (E), and spike (S) proteins [4]. The disease has many pathological effects on vital organs that lead to low egg production and quality; the virus also causing renal damage and is considered a predisposing factor for respiratory bacterial infection specially airsacculitis [5]. Vaccination failure against IBV frequently occurs due to the genetic material evolution of the virus [6]. Many serotypes were identified including Massachusetts (Mass)-type [7] and QX genotype strains [8]. Genotypes and variants added a huge data impact on the genetic diversity of IBV [9]. Lack of cross-protection between IBV genotypes and emerging of new genotypes was always a problem in controlling and preventing the disease [5,10,11]. Several methods used for diagnosis of IBV, including the reverse transcriptase-polymerase chain reaction (RT-PCR) which consider a useful tool in comparing with serological methods [12].

Untranslated regions (UTR), which have high identification characteristics in reverse of the S1 region of the IBV genome, are used for rapid detection and classification for several IBV strain by generating PCR products ranged from 200 bp to 433 bp [13]. The hypervariable region of UTR that includes conserved flanking regions is the main characteristic feature of this region used as a diagnostic tool among different IBV strains [14]. Although this region used in differentiation between two strains, it is also used to show the similarity between other avian species viruses depending on UTR. Leghari *et al*. [15] showed that turkey coronavirus has a resemblance more than 78% when it compared with IBV. UTR also harbor the structural elements involved in replication and translation [16].

This study aimed to diagnose of IBV in suspected farm animals and to prevail the pathological effect of the virus on tracheal and renal tissue.

## Materials and Methods

### Ethical approval

This study does not require ethical approval. However, all applicable international, national, and institutional guidelines for the care and use of animals were followed during the sample collection. 

### Samples collection and histological preparation

In January 2019 tissue samples were collected from the suspected farm during IBV outbreak at Tikrit city. Kidneys collected from 20 suspected animals transported in the icebox to Laboratories of Tikrit University, piece of each sample stores at −85°C for molecular investigation, while the whole samples kept in 10% formalin 24 h before it was placed in 70% ethanol and sent to laboratories for routine histological preparation [17]. The samples that showed positive IBV in the PCR test were sent for further pathological study while negative samples were neglected.

### Viral RNA extraction

Total RNA extracted from animal tissue using QIAzol^®^ (Germany) reagent, 100 mg of tissues cut into pieces with surgical blades and homogenized using of liquid nitrogen, procedure preceded, according to the manufacture instructions. RNA dissolved in 50 μL DEPIC treated water and stored at 85°C until the time of use.

### Preparation of cDNA

TonkBio™ First-strand cDNA synthesis Kit (USA) used to prepare cDNA, 5 μg of total RNA added to 1 μL of 20 pmol of OligodT primer, and 1 μL of 20 pmol of random primer and the volume completed up to 12.5 μL with RNase free water, after incubation at 65°C for 5 min the mixture chilled in ice, spine down and the vial placed back on ice. 4 μL of 5× reaction buffer, 0.5 μL of RNase inhibitor, 2 μL of dNTP mix, and 1 μL TonkBioTM M-MLV (200 U/μL) added. The components mixed gently and spinned briefly then incubated at 25°C for 5 min, followed by 42°C for 1 h. The reaction was terminated by heating at 70°C for 5 min and samples kept at −85°C until time of use.

### Detection of Virus by UTR region amplification

Pair of primers All 1-F 5`-CAGCGCCAAAACAACAGCG-3`, Del1-R 5`-CATTTCCCTGGCGATAGAC-3` used to detected IBV in suspected samples of chicken renal tissue using conventional RT-PCR amplifying segments ranges 200-433 pb. This primer identifies IBV strains targeting the most hypervariable region with conserved flanking regions in the IBV genome [13]. 10 pmol of each primer added to the cDNA (100 ng/μL) in the premix (Bioneer®-korea) tubes and final volume completed up to 20 μL with distilled water. PCR carried out with thermocycler in which DNA denatured for 2 min at 94°C, flowed by 35 cycles of denaturation 45 s at 94°C, annealing 45 s 55, extension 45 s at 72, and a final single cycle of extension for 5 min at 72°C. DNA electrophoresis was subjected using 2% agarose gel, which was photographed using of Gel-documentation system.

## Results

**Figure-1:**
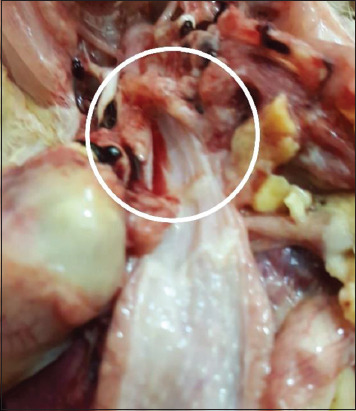
Recent mortal chick showed blockage of tracheal bifurcation by abscess leaked from one sectioned branch.

Chickens of the affected farms showed signs of sneezing and lacrimation with a morbidity rate of 85% and a mortality rate of 20% at 20-day olds ages. Postmortem lesions appeared as tracheal abscesses at the bifurcation site (Figure-1). This study was performed to investigate any potential changes caused by the virus in the trachea and kidney and to investigate the presence of IBV by depending on the UTR site that amplified by specific primer designed to differentiate among IBV strains.

**Figure-2:**
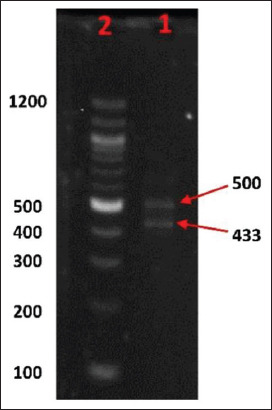
Agarose gel electrophoresis of PCR product; Len 1 Two IBV strain PCR product 500 bp and 433 bp, Len 2, Molecular weight marker 100-bp Ladder.

Molecular results of renal tissue were subjected to a two-step RT-PCR test showed positive confirmation of IBV. The successful amplification of two fragments with a PCR product size of 433 bp and 500 bp (Figure-2) provides evidence for the presence of a virus genome but not determine the time of infection.

**Figure-3:**
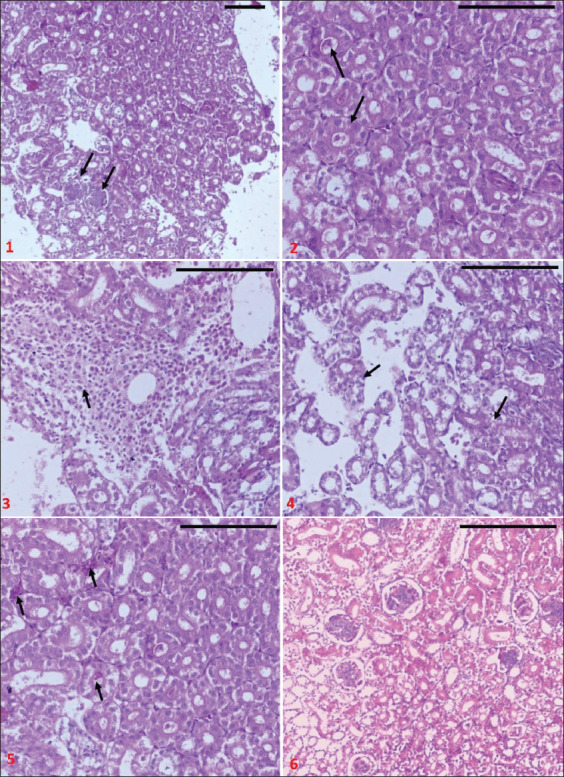
Kidney of broiler chicken infected with IBV, 1: Degenerated glomeruli 2: swelling of tubular epithelial cells with narrowing lumen, 3: Infiltration of inflammatory cells, 4: Necrosis, sloughing and fibrosis of renal tubules endothelia, 5: Renal interstitial congestion, 6: Normal tissue (Hematoxylin and Eosin staining; bar=100 μm).

Histopathological study of renal tissue (Figure-3) showed signs of inflammation and cellular degeneration. The signs included infiltration of neutrophils, disruption of glomeruli, different stages of corruption to the renal tubules. The renal tubules conducted a cellular swelling and narrowing of the lumen with cellular necrosis. In some locations, tubular endothelial cells were replaced by fibrin.

**Figure-4:**
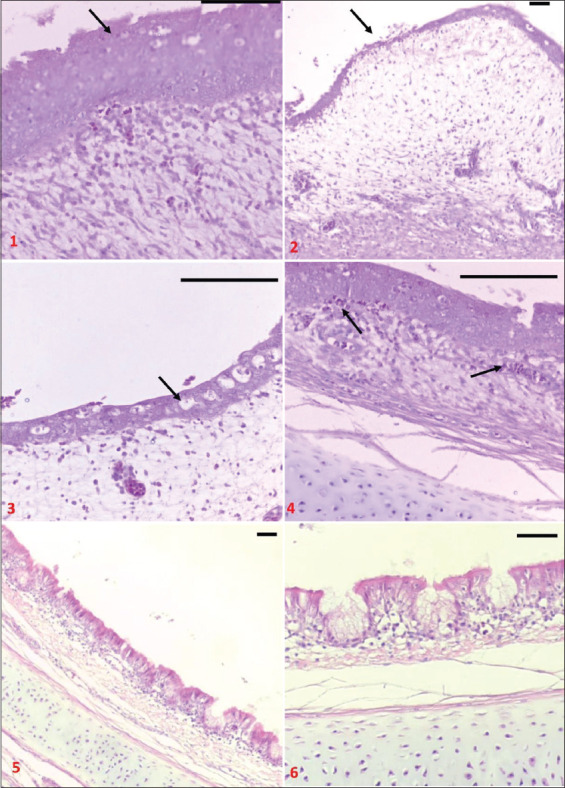
Cross-section in trachea of broiler chicken infected with IBV, 1: Hyperplastic thickening of the epithelial layer, 2: Loss of epithelial layer with cilia over proliferated sub-epithelial layer, 3: Replacement of goblet cells by vacuoles, 4: Sub-epithelial congestion, 5 and 6: Normal tissue (Hematoxylin and Eosin staining; bar=100 μm).

Pathological study to the tissue of the trachea (Figure-4) reveled a remarkable effect of IBV on the mucosal and submucosal area of the trachea. These changes included loss of epithelial cells with its cilia, sever hyperplasia leads to thinking in large epithelial areas, infiltration of neutrophils, and disappearances of goblet cells, which replaced by empty vacuoles with an increased amount of fibrin and congestion in the sub-epithelial layer.

## Discussion

The viral mutation may contribute to different pathological, immunological, replication, and tissue tropism properties [18]. The molecular results suggest that there was one new genotype of IBV not responding to the vaccination process in tested samples, according to the original primer paper [13]. Primer designers named three genotypes to amplify product size of 433 bp were Vaccine A (vaccine strain A Fort Dodge Australia Pty Ltd.), V1/02 (GenBank accession number: FJ235194), and N1/03 (GenBank accession number: FJ235190). The results of the current study showed a new fragment (500 bp) not found in Hewson *et al*. [13], results adding new probable genotype (nucleotides) to the range of UTR primer used in differentiation between IBV strains. The region of interest (UTR) could produce the same amplification product when the strains were similar; otherwise, the UTR sequence could differ from other strains by insertion or deletion nucleotides make a different size of PCR product [19]. Genomic mutations were evidenced in the UTR region of IBV [20]. Researchers revealed that the UTR region contains hypervariable area vary from between IBV strains and may reduce the capacity of the IBV for adsorption [21]. Researchers also recommended not to use the UTR region with S1 gene sequences in phylogenic classification because there is no correlation between IBV strains genome in the UTR region and S1 gene [20].

These changes agree with other researchers’ results, who suggested that the lesions appeared as a result of the apoptotic effect of IBV on cells [22]. The current study results also agreed with other groups of researchers who showed that the IBV causes cellular necrosis and damage after it replicates inside the cells [23,24]. Group of Korean researchers agreed with the current study results of renal tubular degeneration and acute necrosis of epithelial layer of the trachea, and the Korean researchers concluded that the high inflammatory response induced by cytokines during IBV infection attributed in local renal tissue damage [25]. Researchers who agreed with current study results showed that tissue damage occurred by IBV infection corresponded to the immunological effect of inflammatory mediators from stimulated immune cells, primarily by granzyme and interferon-gamma produced by Natural Killer Cells [26]. Grgić *et al*. [27] suggest that the IBV is well associated with different stages of trachea hyperplasia. Other researcher showed that some of IBV strains cause tissue damage not only in the respiratory tract but it also causes tissue damage in both renal and tracheal tissues [28].

## Conclusion

The mortality in affected chickens was caused by respiratory distress. The vaccination at 1-day-old did not protect agents’ current IBV. Furthermore, we conclude that the virus has an affinity for respiratory and renal tissues. A new probable genotype may emerge.

## Authors’ Contributions

All authors participated in the design of the research. MKS: Provided samples and made the prediagnosis to the suspected cases with IIH. STR and IIH: Designed and analyzed the molecular part. NAJ: Histopathological processing was performed and analyzed. All authors drafted, revised and approved the final manuscript.
